# Effects of TC4 Thickness on the Penetration Resistance Behavior of Ti-Al_3_Ti Metal–Intermetallic Laminated Composites

**DOI:** 10.3390/ma18081846

**Published:** 2025-04-17

**Authors:** Yang Wang, Meini Yuan, Pengfei Zhou, Xin Pei, Wei Yang, Zehui Tian

**Affiliations:** 1 School of Aerospace Engineering, North University of China, Taiyuan 030051, China; wy2843000804@163.com (Y.W.); px2565586030@163.com (X.P.); yangwei980107@163.com (W.Y.); 2 School of Mechanical and Electrical Engineering, North University of China, Taiyuan 030051, China; zpf97zpf@163.com; 3 School of Chemistry and Chemical Engineering, North University of China, Taiyuan 030051, China; tzh1950228407@163.com

**Keywords:** impact, Ti-Al_3_Ti, metal–intermetallic laminate composites, finite elements analysis, failure mechanism

## Abstract

Ti-Al_3_Ti metal–intermetallic laminate (MIL) composites with microscale layer thickness have attracted attention in aerospace applications. However, whether millimeter-thick Ti layers can enhance the anti-penetration of Ti-Al_3_Ti MIL composites under 400–1000 m/s impact velocities remains unclear. In this study, a Ti-Al_3_Ti MIL composite target was prepared by hot press sintering, and the 2D finite element model validated by anti-penetration testing was used to prove that increasing the thickness of the Ti layer significantly increases the stress level and anti-penetration limit of the target. Simulations show that compared with a 0.1 mm Ti layer, a 2.5 mm Ti layer reduces the projectile residual velocity by 100% (600 m/s), 72% (800 m/s), and 38.5% (1000 m/s). With a total thickness difference of 0.1 mm, the crack propagation angles increase by 4° (0.06 mm Ti) and 14° (2.5 mm Ti) compared to a 0.4 mm Ti layer. By analyzing stress wave propagation and energy absorption during penetration, this work reveals that millimeter-thick Ti layers improve anti-penetration performance by controlling heterogeneous interface failure and the crack propagation direction through increased ductile layer thickness. These findings provide data for MIL composites and offer potential cost reductions for high-performance anti-penetration materials.

## 1. Introduction

In aerospace and defense applications, the concurrent optimization of lightweight design and high impact protection has long been a core objective. Ti-Al_3_Ti metal–intermetallic laminate (MIL) composites, characterized by high strength, toughness, and low density, have emerged as promising novel lightweight armor materials [1,2,3,4]. The anti-penetration performance of laminated composites is closely linked to the thickness of the constituent layers; however, existing studies primarily focus on the interfacial strengthening effects of micron-scale Ti layers (e.g., 10–100 μm) [1,2,3,4]. The failure mechanisms and energy dissipation behaviors of millimeter-scale Ti layers (0.5–10 mm) under high-velocity impacts (400–1000 m/s) remain largely unexplored. Such multi-scaling thickness differences may induce critical mechanical behavior changes, including alterations in stress wave and crack propagation.

Extensive experimental and numerical studies on the anti-penetration performance of Ti-Al_3_Ti laminates have been conducted globally in recent years. Guo et al. demonstrated that gradient nanocrystalline structures can enhance the anti-penetration limit velocity of Ti-based materials [5]. Ma [6] deduced through numerical simulations that Ti-Al_3_Ti-Al laminated composites exhibit excellent compressive strength and failure strain when the Al content is between 10% and 15%. Laminated composites without Al displayed brittle fracture, with a fracture angle of approximately 45°. Li et al. [7] obtained the optimal layer thickness ratio of Ti-Al_3_Ti-Al as 3:14:4 using genetic algorithms. Yuan et al. [8] studied the influence of ductile Al on the penetration resistance of Ti-Al_3_Ti laminated composite through experimental and numerical simulation methods and believed that ductile metal can improve the penetration resistance by enhancing the toughness of the composite. Cao et al. [9] compared the penetration resistance of monolithic Al_3_Ti and Ti-Al_3_Ti laminated composites by numerical simulation, and the latter had better penetration resistance. The results showed that when cracks propagated from the Al_3_Ti matrix to the interface between the matrix and the reinforcement, the bridging effect of the Ti caused a change in crack direction, and the length of the crack paths resulting from crack deflection and branching increased, leading to the target absorbing more energy. Liu et al. [10] conducted impact tests on Ti-Al_3_Ti laminated composites and applied the Buckingham pi Theory to derive the functional relationship between the target critical velocity, thickness, and the projectile diameter. Thiyaneshwaran et al. [11] investigated the effect of the Ti volume fraction on the density of Ti-Al_3_Ti MIL composites. Zelepugin [12,13] reported that thicker metallic layers at the submillimeter scale can hinder brittle crack propagation. R R. A [1] studied the effect of the Ti volume fraction on the fatigue crack propagation in Ti-Al_3_Ti laminated composite, and the increase in Ti layers improved the fatigue properties of Ti-Al_3_Ti laminated composite. Khan [14] demonstrated that the contact duration under impact loading, interfacial delamination between metal-composite, and metallic shear failure facilitate energy absorption, greatly enhancing the damage resistance under high-stress impacts. Vecchio [15] reported that Ti-Al_3_Ti exhibits excellent anti-penetration performance, with micron-scale Ti layers deflecting crack propagation. I.A. Bataev [16] successfully fabricated Ti-Al_3_Ti laminates with superior interfacial properties by explosive welding.

Although some studies have been conducted on the effect of ductile metal layers on the mechanical properties of laminated composites through experiments and simulations, a few issues still need to be paid more attention. In particular, (i) The influence of ductile metals with millimeter layer thickness on the anti-penetration properties of laminated composites during penetration should be analyzed along with a failure analysis of the laminated composite target. The reason is that in the materials studied by predecessors, the layer thickness scale is mainly concentrated between the micron and sub-millimeter ranges. Even if there are individual cases reaching the millimeter scale, only the role of brittle materials in the anti-penetration process is focused on. In our recent experiments, it has been shown that the mechanical properties of laminated composites with millimeter thicknesses of elements are better than those of micron and sub-millimeter thicknesses of elements paired with laminated composites. (ii) The interface behavior and failure mechanism of MIL composites under different penetration velocities have not been systematically studied yet. In addition, based on our previous research on the Ti-Al_3_Ti MIL composite target plate, we note that the performance variation with respect to the thickness of Ti is comparable to some characteristics observed in heterostructures [17]. Existing research results of heterostructures have provided valuable insights into the mechanical behavior influenced by layer thickness [18,19,20].

To address the above issues, this study systematically investigates the effect of Ti layer thickness ranging from the micron to millimeter scales on the anti-penetration performance of Ti-Al_3_Ti MIL composite target plates within the impact velocity range of 400–1000 m/s. By validating a finite element model for Ti-Al_3_Ti MIL composites through anti-penetration tests, the research aims to (1). quantify the relationship among Ti layer thickness, projectile residual velocity, and energy absorption; (2). characterize the interfacial failure modes (such as crack deflection and stress wave dissipation) of the Ti-Al_3_Ti MIL composite target plates with millimeter-thick Ti layers under different velocities; and (3). propose design criteria for optimizing the thickness of the ductile layer to enhance the anti-penetration performance.

## 2. Materials and Methods

### 2.1. Material Preparation

**Figure 1:**
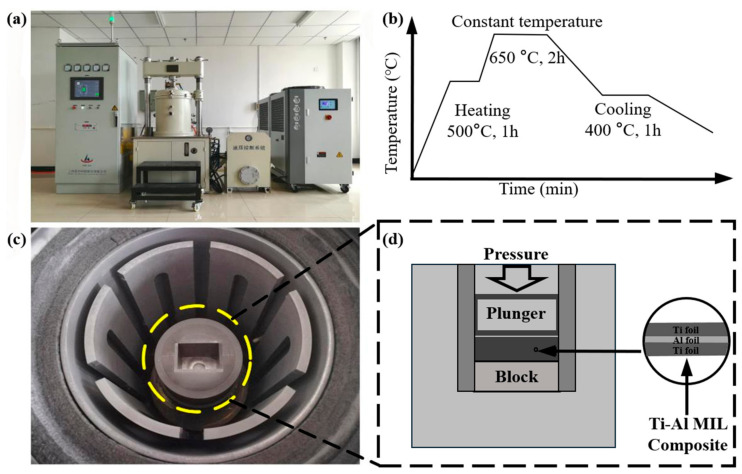
(**a**,**c**) Sintering furnace, (**b**) hot-pressing sintering process, (**d**) schematic of hot-pressing process.

The preparation process of the Ti-Al_3_Ti target includes two steps: pretreatment and heat treatment. In the pretreatment step, Ti-6Al-4V (Ti) with a thickness of 100 µm and aluminum (Al) foil are cut into 100 mm × 100 mm rectangles. Table 1 presents the chemical compositions of each metal foil. Then, they are etched separately in designated solutions containing 87% nitric acid + 10% HF + 3% water and 20% sodium hydroxide. In the heat treatment step, Ti and Al foil are alternately stacked to form a laminated structure. Subsequently, they are sintered in a vacuum hot press (ZT-40-21Y). Specific manufacturing parameters, including the reaction temperature, pressure, and processing time, are shown in Figure 1.

### 2.2. Anti-Penetration Experiments

**Figure 2:**
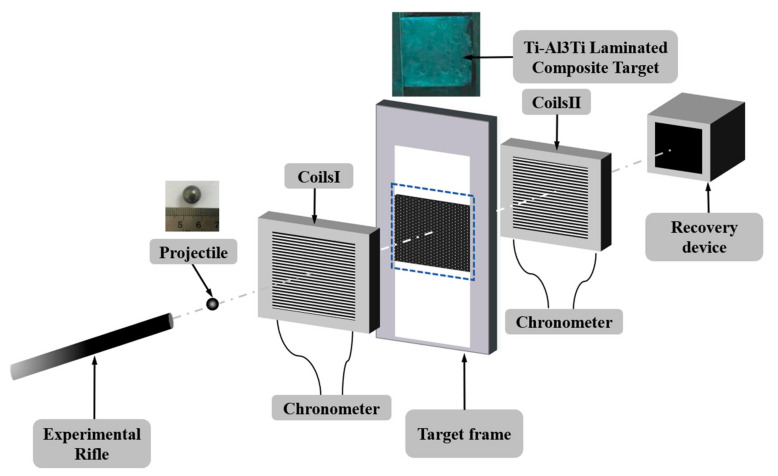
Schematic diagram of the anti-penetration experiment.

In this study, penetration tests of the Ti-Al_3_Ti target were conducted according to the GA 950-2019 testing standard [21]. The experimental setup included a test rifle, projectiles, two velocity measurement coils and a chronometer, target supports, and projectile recovery devices. Specifically, a 12.7 mm rifle was used, with a firing distance of 6 m. The projectiles were tungsten alloy spherical fragments with a diameter of 9.45 mm and a mass of 8.1 g. The specific principles of penetration testing are shown in Figure 2. The projectiles were fired vertically at the target using the test rifle at a certain velocity controlled by the amount of gunpowder. A velocity measurement coil was placed in front and behind the target support, connected to a timer to record the initial and remaining velocities of the projectiles. The support was fixed on both sides of the target to position it in the middle of the support. A recovery device was used to collect the remaining projectiles after piercing the target and fragments from the back of the impacted target.

The dimensions of the Ti-Al_3_Ti target and experimental penetration velocity are 100 mm × 100 mm × 10 mm, 364 m/s, 388 m/s, 485 m/s, and 533 m/s, respectively.

### 2.3. Finite Element Simulations

**Figure 3:**
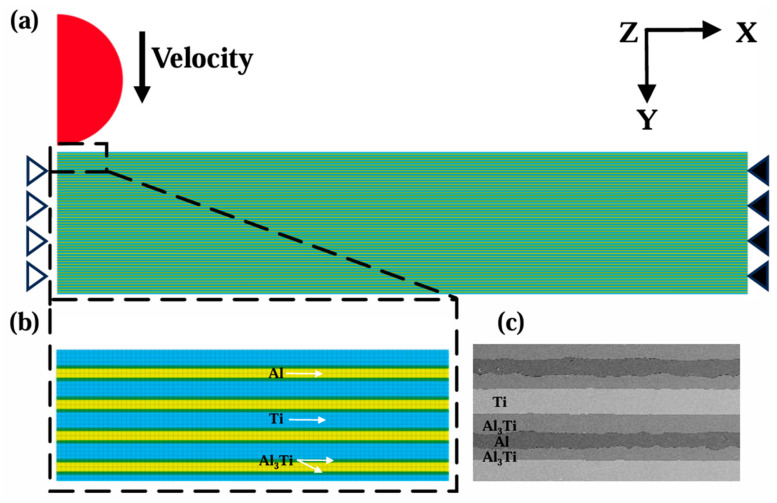
(**a**) Finite element model of the projectile and Ti-Al_3_Ti MIL composite and boundary constraints. (**b**) Schematic diagram of the mesh partitioning. (**c**) Microstructure of Ti-Al_3_Ti MIL composite.

This paper analyzed and quantified the microstructure of Ti-Al_3_Ti laminated composite materials. Corresponding 2D finite element models were established (Figure 3), and analyses were conducted using the finite element software Ls-Dyna 19.0. The material composition of the model is shown in Table 2.

To improve computational efficiency, a two-dimensional axisymmetric finite element model of Ti-Al_3_Ti was established based on the symmetrical characteristics of the target. The projectile had a radius of 4.75 mm, while the target was 50 mm long and 10.3 mm thick. PLANE162 elements were utilized for meshing, and a volume-weighted Lagrangian algorithm was employed to calculate the projectile and target. Mapped meshing was employed to ensure accuracy and stability in the computations. Grid refinement was performed around the target center to ensure an accurate representation of the complex interactions between the projectile and target. In addition, in mesh sensitivity studies, an excessive amount of mesh could not improve the accuracy of the calculations, and the sacrifice is a longer computational time. Finally, the minimum mesh size was 0.003 mm × 0.003 mm, and the element number of the target was 219800.

In interface constraints, the model employed was a common-node method to describe the strong bonding interface in Ti, Al_3_Ti, and Al within the Ti-Al_3_Ti target. In order to avoid mesh penetration of the projectile–target contact model, a two-dimensional single-sided penalty function algorithm was employed in this model.

In boundary constraints, non-reflecting boundary constraints were placed at the radial boundaries of the target material. Additionally, fixed constraints were applied to the edges of the composite layers, and constraints on y-direction rotations and displacements in the x and z directions were applied to the symmetry interface of the model (Figure 3).

### 2.4. Constitutive Models and Parameters

In numerical simulations, the accuracy of the simulation results is determined by the material constitutive model, so the choice of material model is a key issue. In this study, the projectile target system mainly consists of tungsten alloy, Ti, Al, and Al_3_Ti, among which tungsten alloy, Ti, and Al were used for the *MAT-JOHNSON-COOK (J-C) constitutive model [22], Johnson–Cook dynamic failure model, and *EOS Gruneisen equation of state. Specific material parameters are provided in Table 3. The Johnson–Cook (J-C) model is a phenomenological model commonly used to predict the material response of metals under high strain rates and impact loads, and the J-C constitutive model explicitly considers the effects of strain, strain rate, and temperature. In addition, the Johnson–Holmquist (JH-II) [23] constitutive model is used to describe the stress–strain behavior of brittle materials such as Al_3_Ti, with specific parameters listed in Table 4. In general, ceramic materials are inherently brittle. They have high compressive strength but low tensile strength, and due to the growth of microcracks, they often exhibit progressive damage under compressive loads. The Johnson–Holmquist material model with damage evolution (JH-II) can be used to simulate the failure of brittle materials subjected to high pressure and high strain rates.

The simulations were conducted using a commercial explicit code LS-DYNA (Livermore Soft Technology Corporation, Livermore, CA, USA). According to the Johnson–Cook model, the dynamic flow stress is described by the strain, strain rate, and temperature, and the constitutive equation is expressed as Equation (1).

(1) $$\sigma_{y}(\varepsilon_{p},\varepsilon_{p}^{*},T)=[A+B(\varepsilon_{p}{)}^{n}][1+Cln(\varepsilon_{p}^{*})][1-(T^{*}{)}^{m}]$$

*A*, *B*, *c*, *n*, and *m* are custom parameters, $\varepsilon_{p}$ is the equivalent plastic strain, $\varepsilon_{p}^{*}$ is the dimensionless rate of strain. $\varepsilon_{p}^{*}=$ $\varepsilon /\varepsilon_{0}$, where ε is the actual strain rate and $\varepsilon_{0}$ is a reference strain rate.

Gruneisen provides pressure calculation methods for both compression and expansion states. The hydrostatic pressure control equation holds true when *u* ≥ 0. The Gruneisen is expressed as Equation (2).

(2) $$p=\frac{\rho_{0}C^{2}\mu [1+(1-\frac{V_{0}}{2})\mu -\frac{a}{2}\mu^{2}]}{{\left[1-(S_{1}-1)\mu -S_{2}\frac{\mu^{2}}{\mu +1}-S_{3}\frac{\mu^{3}}{(\mu +1{)}^{2}}\right]}^{2}}+(\gamma_{0}+\alpha \mu )E$$

In addition, the Johnson–Cook dynamic failure model characterizes the damage of metals under high strain rate loading. It is based on the equivalent plastic strain values at the integration points of elements. The damage parameter (*ω*) is defined by Equation (3).

(3) $$\omega =\sum \left(\frac{\Delta -\varepsilon^{pl}}{-\varepsilon^{pl}}\right)$$

The plastic failure strain ($\varepsilon_{f}^{p1}$) is given by the following Equation (4):

(4) $$-\varepsilon_{f}^{p1}=[d_{1}+d_{2}exp(d_{3}\frac{p}{q})][1+d_{4}ln(\frac{-\varepsilon^{p1}}{\varepsilon_{0}})][1+d_{5}\frac{\left(T-T_{room}\right)}{\left(T_{m}-T_{room}\right)}]$$

The specific representation is as follows:

(5) $$\sigma^{*}=\sigma_{i}^{*}-D(\sigma_{i}^{*}-\sigma_{f}^{*})$$

$\sigma_{i}^{*}$ and $\sigma_{f}^{*}$ represent the normalized equivalent strengths of the material under undamaged and fully damaged conditions, respectively. They are represented as follows:

(6) $$\sigma_{i}^{*}=A_{r}(P^{*}+T^{*}{)}^{N}[1+C_{r}ln\varepsilon^{*}]$$

(7) $$P^{*}=P/P_{HEL}T^{*}=T/P_{HEL}{\dot{\varepsilon }}^{*}=\dot{\varepsilon }/{\dot{\varepsilon }}_{0}$$

(8) $$\sigma_{f}^{*}=B_{r}(P^{*}{)}^{M}[1+C_{r}ln\varepsilon^{*}]$$

(9) $$\sigma_{f}^{*}\leq \sigma_{fmax}^{*}$$

where $A_{r}$, $B_{r}$, $C_{r}$, *M*, *N*, and *T* represent material fitting parameters. *P* represents the hydrostatic pressure. *T* is the ultimate static tensile strength. $P_{HEL}$ represents the compressive strength of the material under Hugoniot elastic limit. $\dot{\varepsilon }$ represents the strain rate under dynamic loading. ${\dot{\varepsilon }}_{0}$ is the reference strain rate. $P^{*}\;$ is the dimensionless equivalent hydrostatic pressure of the material. $T^{*}$ is the dimensionless equivalent hydrostatic pressure of the material. ${\dot{\varepsilon }}^{*}$ represents the dimensionless equivalent strain rate of the material. $\sigma_{fmax}^{*}$ represents the maximum normalized equivalent fracture strength of the material.

The damage of brittle materials is similar to the expression of the JC model. Its material damage parameter *D* can be expressed as: $D=\sum (\Delta \varepsilon_{p}/\varepsilon_{p}^{f})$*,* where $\Delta \varepsilon_{p}$ represents the cumulative integral of effective plastic strain in a single cycle of material. $\varepsilon_{p}^{f}$ represents the ultimate plastic strain of the material under static hydrostatic pressure *P* and can be expressed as: $\varepsilon_{p}^{f}=D_{1}(P^{*}+T^{*}{)}^{D_{2}}$.

In the JH-II model, a cubic polynomial is primarily used to represent the state equation of the material in the undamaged state.

(10) $$\mu \geq 0:P=K_{1}\mu +K_{2}\mu^{2}+K_{3}\mu^{3}$$

(11) $$\mu <0:P=K_{1}\mu$$

where *P* is the hydrostatic pressure. $K_{1}$ is the volume modulus. $K_{2}$ and $K_{3}$ are material parameters. *μ* is the bulk strain and can be expressed as: $\mu =\rho /\rho_{0}-1$. ρ is the instantaneous density at a certain hydrostatic pressure, and $\rho_{0}$ is the original density of the material. As the material damage increases, the volume of the material expands, introducing an incremental: ΔP

(12) $$\mu \geq 0:P=K_{1}\mu +K_{2}\mu^{2}+K_{3}\mu^{3}+\Delta P\mu$$

Assuming that the energy loss of the material is converted into fluid static potential energy, then the energy conversion equation can be approximated as follows:

(13) $$(\Delta P_{t+\Delta t}-\Delta P_{t})\mu_{t+\Delta t}+(\Delta P_{t+\Delta t}^{2}-\Delta P_{t}^{2})/2K_{1}=\beta \Delta U$$

In the above equation, β represents the energy conversion coefficient, ranging from 0 to 1, and ΔU represents the energy loss.

A is the intact normalized strength parameter, B is the fractured normalized strength parameter, C is the strength parameter (for strain rate dependence), M is the fractured strength parameter (pressure exponent), *n* is the intact strength parameter (pressure exponent), T is the maximum tensile pressure strength, and $D_{1}$ and $D_{2}$ are parameters for plastic strain to fracture.

## 3. Results and Discussion

### 3.1. Model Validation

**Figure 4:**
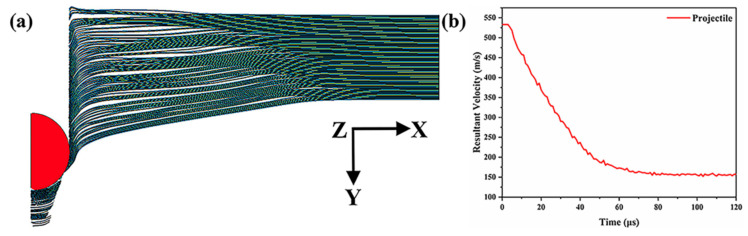
(**a**) Simulation result and (**b**) residual velocity–time relations of projectile penetration of the Ti-Al_3_Ti targets containing Ti with a 0.1 mm thickness with 533 m/s initial velocity.

Numerical modeling of the microstructure of Ti-Al_3_Ti MIL composites was conducted in this study, followed by penetration simulations under the same experimental conditions, and the simulation results were compared with penetration experimental results, as shown in Table 5. When the projectile penetrated the Ti-Al_3_Ti target at a velocity of 533 m/s, the experimental residual velocity of the projectile was 162 m/s. Subsequently, the damage results were compared using Ls-Prepost 4.5 software. The simulation result was 157 m/s, and results are shown in Figure 4. This result is within the allowable error, and the models used in this paper can accurately predict the anti-penetration effects of the Ti-Al_3_Ti targets.

### 3.2. Anti-Penetration Property of Material

Based on the results of Section 3.1, we conducted corresponding finite element simulations for the Ti-Al_3_Ti MIL composite targets with different Ti layer thicknesses to explore the influence of the variation in Ti layer thickness on the anti-penetration performance of Ti-Al_3_Ti MIL composites under different impact velocities. The Ti layer thicknesses examined were 0.1 mm, 0.2 mm, 0.4 mm, 0.6 mm, 0.8 mm, 1 mm, 2.5 mm, and 5.1 mm. The initial velocities of the projectile were 400 m/s, 600 m/s, 800 m/s, and 1000 m/s, and the numerical simulation results are shown in Table 6.

Table 6 shows that the residual velocity of the projectile decreases with the increase in Ti layer thickness. When the Ti layer thickness is 2.5 mm, this value reaches the minimum. This means that increasing the layer thickness of ductile Ti from the micron level to the millimeter level can enhance the anti-penetration performance of the Ti-Al_3_Ti MIL composite, and this trend has similar results under other penetration velocities. The reason is that the relatively thick Ti layer can absorb more energy through mild elastoplastic deformation while weakening the intensity of the stress wave reaching the heterogeneous interface, avoiding the interface damage caused by the tensile failure of Al_3_Ti, and dispersing the stress concentration during the impact process. In addition, because Ti and Al_3_Ti have good strain rate sensitivity [20], with the increase in the penetration velocity, the yield strength of each layer of material in the target plate is higher, the penetration resistance to the projectile is greater, and the synergistic effect of the heterogeneous interface is also stronger. This is consistent with the research results of Yuan [8] and Li [24].

**Figure 5:**
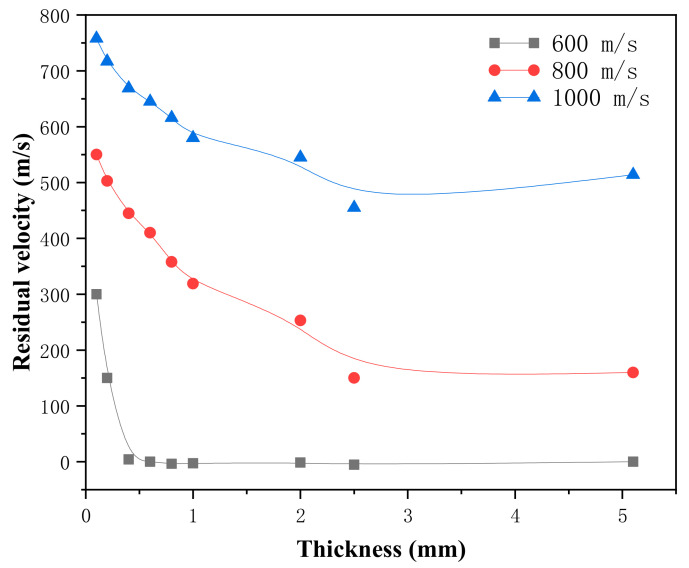
Residual velocity–thickness history of the projectile penetrating the Ti-Al_3_Ti MIL target at different velocities.

**Figure 6:**
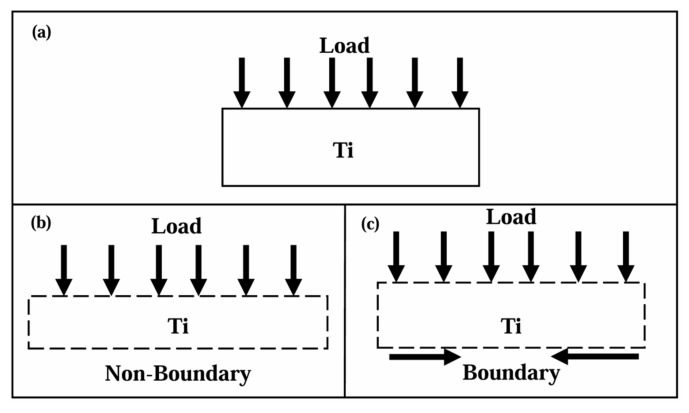
Schematic diagram of heterogeneous structure deformation: (**a**) single-layer target, (**b**) without boundary constraint, (**c**) boundary constraint.

In order to further study the influence of the layer thickness on the anti-penetration performance of the target plate, we compared the velocity–time histories at different penetration velocities as shown in Figure 5. In Figure 5, with the increase in the Ti layer thickness, the decreasing trend of the residual velocity of the projectile becomes moderated. With the increase in the penetration velocity, the optimal Ti layer thickness also gradually increases, and when the Ti layer thickness is 2.5 mm, the residual velocity of the projectile is the smallest and the anti-penetration performance of the target plate is the best, as shown in Figure 5. This indicates that the layer thickness of the Ti layer has a great impact on the anti-penetration performance of the target plate. Attention should be paid to the fact that the anti-penetration property of the Ti-Al3Ti MIL composite target is not positively correlated with the thickness of the Ti layer. For example, when the penetration velocity is 1000 m/s and the Ti layer thickness is 5.1 mm, the residual velocity of the projectile is not the lowest. This is because in the laminated composite, the synergistic effect of the heterogeneous interface also deeply affects the mechanical properties of the layered composite. The mutual constraint of the main tough-brittle materials changes the loading form of the MIL composite, as shown in the schematic diagram in Figure 6. Under the constraint of the heterogeneous interface on Ti, the Ti layer produces smaller strain and obtains a higher strength at the same time. Since this is consistent with the research of Li [25], it also shows that the role of the heterogeneous interface in the anti-penetration process of the MIL composite cannot be ignored. The research of Huang and Su [18,26] shows that the influence of this interface synergistic effect on the dynamic mechanical properties of the heterogeneous structure in the thickness direction is at the micrometer scale. In general, in this study, the factors affecting the anti-penetration performance of the target are the layer thickness of the Ti layer and the synergistic effect of the heterogeneous interface, among which the influence of the Ti layer thickness is the dominant factor. The optimal thickness of Ti increases with the increase in the penetration velocity, and the residual velocity of the projectile first increases and then decreases with the increase in the Ti layer thickness.

### 3.3. Influence of Ti Layer Thickness on Penetration Process

**Figure 7:**
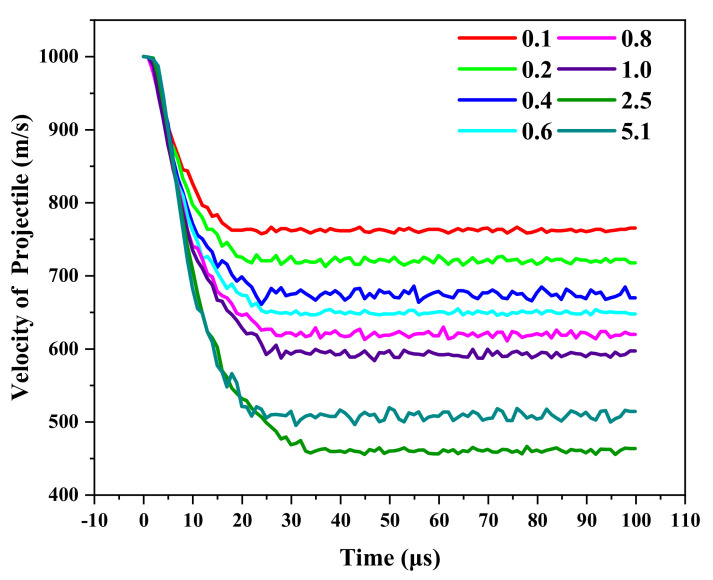
Velocity-Time relations of projectile penetration into Ti-Al_3_Ti target with different Ti thicknesses (mm) at 1000 m/s.

**Figure 8:**
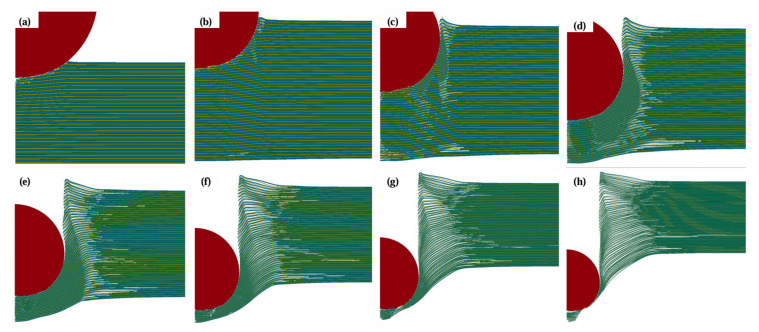
Penetration process of the Ti-Al_3_Ti MIL composite target with Ti thickness of 0.1 mm, in which a tungsten alloy projectile with an impact velocity of 1000 m/s was employed in FEA: (**a**) t = 2 μs, (**b**) t = 5 μs, (**c**) t = 7 μs, (**d**) t = 10 μs, (**e**) t = 13 μs, (**f**) t = 17 μs, (**g**) t = 20 μs, (**h**) t = 24 μs.

To further study the influence of the Ti layer thickness on the anti-penetration performance of the MIL target, we described the mechanical characteristics of different stages of the projectile penetrating the Ti-Al_3_Ti MIL composite target by analyzing the changes in the projectile velocity, acceleration, and stress state with time. In Figure 7, according to the velocity and slope of the projectile, the penetration process is divided into three stages. These stages include the cratering stage, the stable penetration stage, and the rebound stage [27]. Due to the different penetration velocities and anti-penetration performances of the MIL targets, there are some differences in the time intervals of the penetration stages of different targets. Since the velocity change trend of the projectile is the same, this section takes the penetration of the target with a Ti layer thickness of 0.1 mm at 1000 m/s as an example to describe the penetration process. The penetration process of the projectile is shown in Figure 8.

#### 3.3.1. Cratering Stage

**Figure 9:**
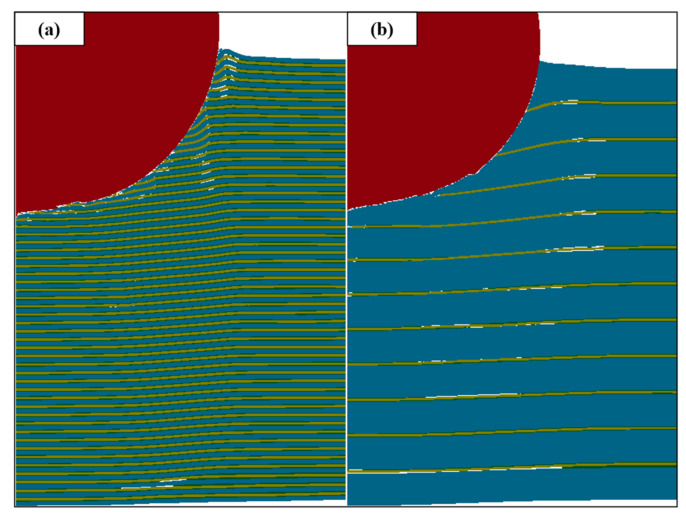
Damage distribution of the target with a Ti thickness of 0.1 mm (**a**) and 0.8 mm (**b**) at 5 μs.

The first stage is the cratering stage, which is between 0 and 5 μs as shown in Figure 8a,b. At this stage, the instantaneous impact of the projectile and the target releases a large amount of energy and rapidly reduces the velocity. At the moment when the projectile and the target contact, the front surface of the target undergoes severe compressive deformation and failure, and the energy of the projectile is divided into internal energy and kinetic energy. The material at the front of the projectile simultaneously produces compressive failure of Ti and Al and impact fragmentation of Al_3_Ti at an extremely high strain rate, which is represented by the deletion of elements in Figure 8. As the projectile continues to penetrate, the projectile produces lateral extrusion on the target, and the Ti and Al layers produce different degrees of shear strain and bending deformation, causing many microcracks and local failure delamination in the thickness direction of Al_3_Ti due to compressive shear as shown in Figure 9a. Subsequently, under the action of compressive load, the Ti layer and the Al layer quickly recontact, the impact stress is redistributed, and the stress intensity of each layer of the target is reduced. At the same time, in the cratering stage, local cracks also appear on the back surface of the target, mainly manifested as the failure of Al_3_Ti, and the main failure reason is that the stress wave reflects at the free interface to produce a large tensile wave and tensile displacement.

**Figure 10:**
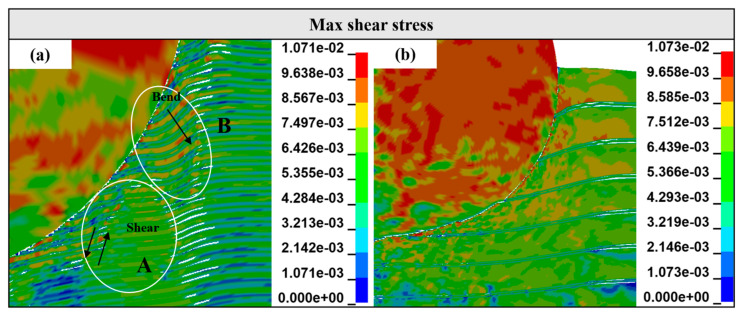
Maximum shear stress nephogram around the projectile with a Ti thickness of 0.1 mm (**a**) and 0.8 mm (**b**) at 5 μs.

**Figure 11:**
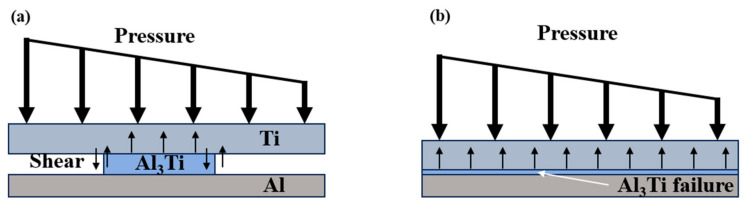
Schematic diagram of heterogeneous structure shear (**a**) and compression (**b**).

It is worth noting that in Figure 9, compared with the Ti-Al_3_Ti MIL composite target with a millimeter-level thickness of the Ti layer, the bending phenomenon of the ductile layer in the target with a micron-level thickness of the Ti layer will be aggravated. This is caused by the combined action of the non-uniform stress transfer caused by the local failure of the brittle Al_3_Ti and the tensile stress wave generated at the free interface, as shown at B in Figure 10a. In the vertical thickness direction, due to the change in the load transfer form, the velocities of the projectile in Ti—0.8 mm and Ti—0.1 mm are 900 m/s and 930 m/s, respectively. In addition, we checked the maximum shear stress nephogram of the target, and the results show that the un-failed Ti–Al_3_Ti structure has a shearing effect on the Ti and Al layers, as shown at A in Figure 10a, which weakens the residual strength of the ductile layer. The schematic diagram of the force on the target is shown in Figure 11. According to the mechanics of the materials, the penetration resistance generated in Figure 11a is lower than that in Figure 11b, because the Ti layer is more likely to break under the multiple actions of shear and compressive loads. In general, the significant increase in the Ti layer thickness improves the anti-penetration performance of the target by changing the adverse effects of the bending stacking and uneven stress distribution of the material in the cratering stage on the anti-penetration performance of the target.

#### 3.3.2. Stable Penetration Stage

**Figure 12:**
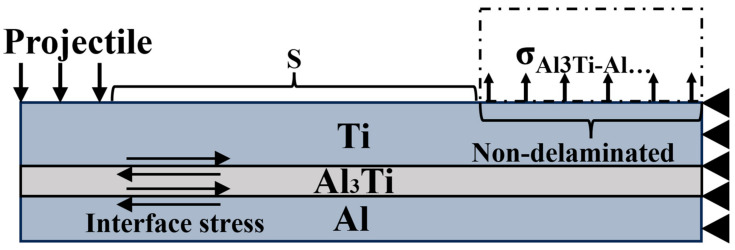
Schematic diagram of the force on the remaining part of the MIL target in the stable penetration stage.

The stable penetration stage occurs within 6 to 25 μs, as shown in Figure 8c,f. At this stage, the projectile enters the target, the lateral compressive effect on the target is reduced, and it is converted to vertical compression and shear damage on the target. At the same time, the stress wave generated by the continuous penetration of the projectile aggravates the delamination failure on the back surface of the target and the transverse expansion of cracks. The main energy absorption methods of the target are the frictional shear between the ductile layer and the projectile, the impact with the remaining target, and the synergistic effect of the heterogeneous interface. In Figure 8f, a large amount of Al_3_Ti around the projectile fails and delaminates, and the target obstructs the projectile through the flexural strength of the un-failed part of the material, the coordination effect of the heterogeneous interface, and the tensile stress provided by the heterogeneous structure. The loading form is shown in Figure 12. Figure 7 shows that the velocity of the projectile changes gently at this stage, and the slope of the curve (acceleration) decreases. Moreover, with the increase in the Ti layer thickness, the duration of the projectile in the stable penetration stage increases, and the velocity reduction is more prominent. This is because the main role of the thin Ti layer is to hinder the crack propagation caused by the failure of Al_3_Ti layers and improve the anti-penetration performance of the target through crack bridging. The thick Ti layer provides higher flexural strength on the basis of hindering crack propagation, increases the friction time between the projectile and the target, and further reduces the velocity of the projectile.

In addition, during the stable penetration process, since the tensile strength of Al_3_Ti is much lower than its compressive strength, the penetration resistance provided by the non-separated interface gradually decreases, and the flexural strength of the remaining material of the target is the main penetration resistance. Since Al_3_Ti absorbs a large amount of impact load and fails and delaminates in the cratering stage, a thicker Ti layer will increase the anti-penetration ability of the target through higher flexural strength, as shown in Figure 12, σ_Al3Ti_ is gradually decreased, and the distance (S) between the load and the delaminated interface is increased. According to the mechanics of the materials, the anti-penetration ability of the target decreases. This tearing delamination phenomenon is the same as that in the research of Yuan [8].

Compared with the thick Ti layer, the thin Ti layer cannot evenly spread the impact load to the Al_3_Ti layer in time, causing the premature failure of part of the Al_3_Ti and reducing the anti-penetration performance of the target.

**Figure 13:**
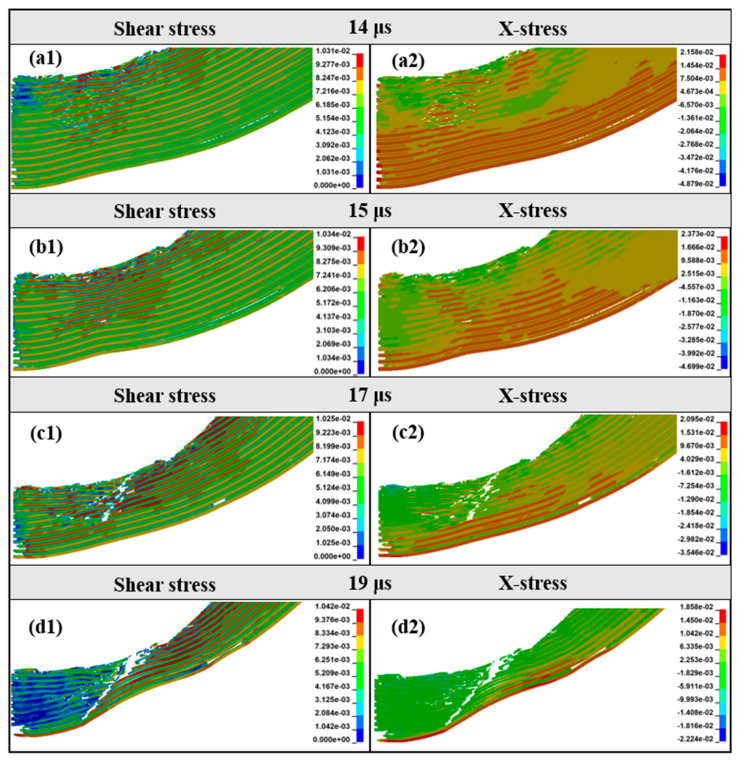
Shear stress and x-direction stress distribution of 14–19 μs punching plug of target plate. Shear stress (**a1**) 14 μs, (**b1**) 15 μs, (**c1**) 17 μs, (**d1**) 19 μs. X-direction stress (**a2**) 12 μs, (**b2**) 13 μs, (**c2**) 14 μs, (**d2**) 19 μs.

**Figure 14:**
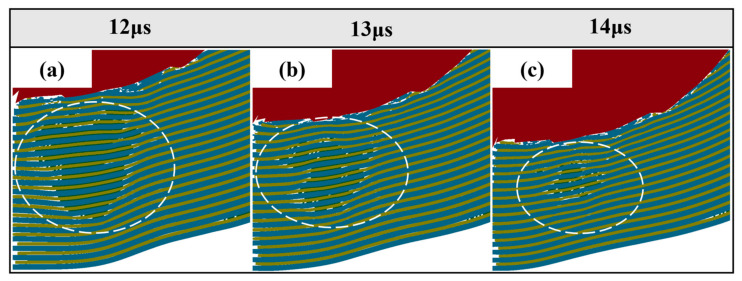
Schematic partial shear. (**a**) 12 μs, (**b**) 13 μs, (**c**) 14 μs.

It can be found from Figure 8f,g that with the penetration of the projectile, the shear punching of the material occurs on the back surface of the target. Figure 13 shows the stress nephogram of the punching process of the target. Obviously, the failure mode of the target around the projectile is shear failure, the back plate is tensile fracture, and finally a trapezoidal punching body is formed. It can be seen from Figure 14 that during this process, part of the Al_3_Ti does not fail, and this part of the laminated structure that did not fail generated the same shear stress on the ductile layer as in Figure 10 in the cratering stage. The fracture strength of part of the ductile layer was reduced, resulting in a decrease in the anti-penetration performance of the target plate. In addition, it is known from the post-processing software that in the stable penetration stage, the stress level of Al_3_Ti near the back plate is 324 Mpa, which is much lower than its compressive limit of 900 Mpa, and the failure reason is interlayer shear failure [28]. This also proves that the mechanical properties of the Ti-Al_3_Ti MIL composite are not fully utilized in the anti-penetration process.

#### 3.3.3. Perforation Stage

**Figure 15:**
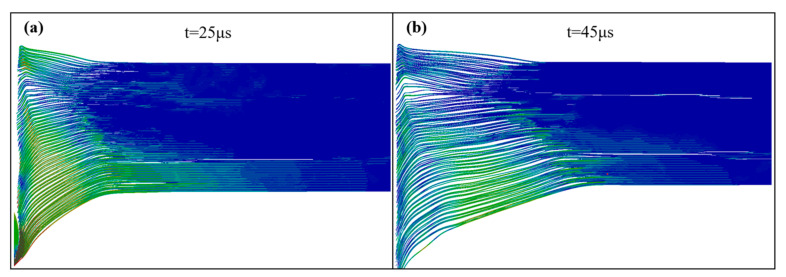
Stress nephograms of the target at 25 μs (**a**) and 45 μs (**b**).

The perforation stage occurs between 25 μs and 45 μs. At this stage, depending on the penetration velocity and the thickness of the Ti layer, the projectile may penetrate, rebound, or embed in the target. Inside the target, with the reflection and dispersion of the stress wave, the internal delamination and crack propagation continue. Comparing Figure 15a,b shows that even after the projectile leaves the target, additional delamination and deformation will occur.

#### 3.3.4. Anti-Penetration Mechanism of Ti-Al_3_Ti MIL Target with Different Thicknesses of Ti Layer

During the penetration process, the loading form of the laminated composite has a great influence on the anti-penetration performance of the target, which is closely related to the thickness of each layer. It can be seen from Figure 7 that with the increase in the layer thickness, the velocity of the projectile decreases more in the cratering stage; in the stable penetration stage, the interaction time between the projectile and the target increases, and the slope of the velocity change increases.

The main reasons for these phenomena are that the increase in the Ti layer thickness improves the bearing capacity of the target in different stages and changes the failure forms of each layer. As a high-strength ductile metal, the increase in the thickness of Ti has the following changes on the anti-penetration behavior of the target: 1. It reduces the bending stack phenomenon of the ductile layer in the cratering stage, making the impact stress transfer more uniform. 2. It enhances the coordinated strain ability of each layer of the target through the strain gradient in the cratering stage and increases the friction time between the target and the projectile in the stable penetration stage. 3. It changes the failure form of more Al_3_Ti at the front of the projectile from shear failure to compressive failure, allowing Al_3_Ti to bear a larger load. By using the post-processing software to extract the stress levels of Al_3_Ti at the same position when the Ti layer thicknesses are 0.1 mm, 0.8 mm, and 2.5 mm and the penetration depths are the same, the results are 445 Mpa, 512 Mpa, and 552 Mpa, respectively, and the stress levels at the moment of impact are 958 Mpa, 932 Mpa, and 1120 Mpa, respectively. The results show that although the content of Al_3_Ti decreases, its load-bearing efficiency increases. In addition, for the projectile penetrating the entire Ti, the residual velocity of the projectile increases. This is because the loss of the layered structure reduces the strength of the target, although the thicker Ti brings better plastic deformation ability.

In general, in the structural design of the Ti-Al_3_Ti MIL composite, the Ti layer with a millimeter-level thickness in the target has more advantages in load transfer and deformation mode than the traditional micron-level thickness and has better anti-penetration performance.

### 3.4. Analysis of Stress Behavior of Ti-Al_3_Ti

During the penetration process, the propagation of the stress wave plays a huge role in the anti-penetration process of the target [29]. Therefore, it is very necessary to discuss the influence of the Ti layer thickness on the propagation of the stress wave during the penetration process.

Through the analysis of the penetration process, it can be seen that the cratering stage, as the time period when the projectile and the target start to contact, has an important influence on the internal stress transfer state and energy absorption mode of the target and the subsequent anti-penetration behavior. The stress states generated by the spherical projectile on each layer of the target during the penetration process mainly include transverse compressive shear stress, compressive stress near the symmetry axis, and tensile stress deviating from the symmetry axis. In the cratering stage, the compressive stress wave generated by the impact of the projectile propagates outward in a circular shape inside the target. When the compressive stress wave encounters a heterogeneous interface, different degrees of tensile and compressive waves are formed through reflection and transmission. According to the stress wave propagation theory, the waveform of the reflected wave is related to the wave impedance between materials. The wave impedance formula is shown in Equation (14). When the incident wave enters a medium with a small wave impedance from a medium with a large wave impedance, the stress of the reflected wave is opposite to that of the incident wave, and the stress of the transmitted wave is the same as that of the incident wave. In other words, if the incident wave is a compressive wave, after passing through the interface from a large wave impedance to a small wave impedance, the reflected wave is a tensile wave and the transmitted wave is a compressive wave.

(14) R=ρC

**Figure 16:**
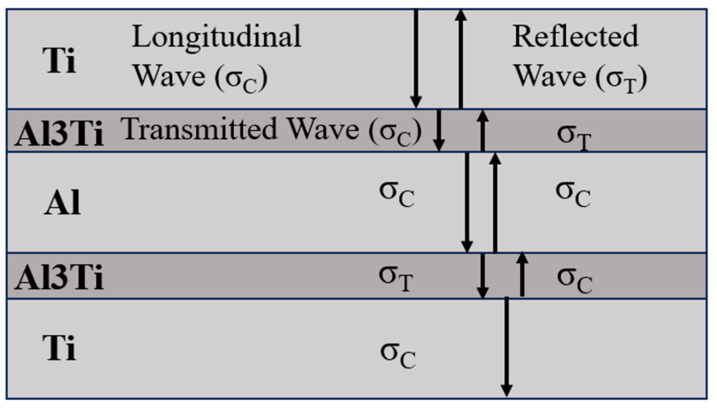
Reflection and transmission of stress waves in Ti-Al_3_Ti-Al. Compression wave (σ_C_), tensile wave (σ_T_).

*R* is the wave impedance, *ρ* is the material density, and *C* is the wave velocity. The wave impedances of the components of the Ti-Al_3_Ti laminated composite are shown in Table 7. According to Table 7, we can confirm that the reflected and transmitted waves at the Ti-Al_3_Ti interface are compressive and tensile stress waves, respectively, and that the reflected wave at the Al_3_Ti-Al interface is a tensile wave, as shown in Figure 16.

**Figure 17:**
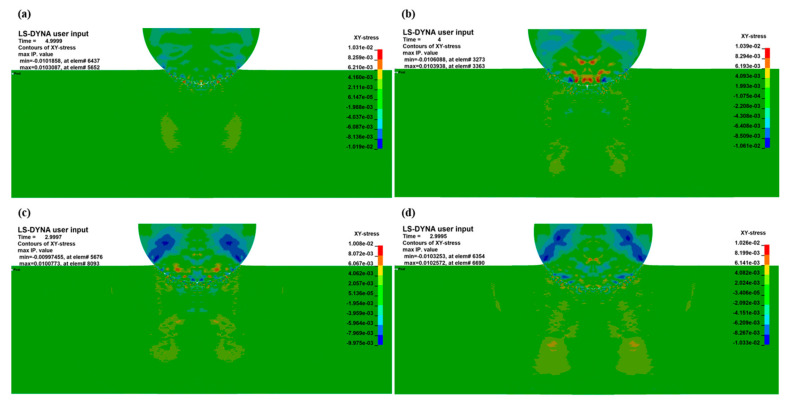
XY-plane stress distribution of a circular projectile with a penetration velocity of 400 m/s (**a**), 600 m/s (**b**), 800 m/s (**c**), and 1000 m/s (**d**) penetrating a Ti layer with a thickness of 0.1 mm.

Due to the large number of interfaces in the laminated material, the stress wave is reflected and transmitted multiple times at the interface, and finally a rather complex stress state is formed inside the matrix. It can be seen from the XY-stress nephogram (Figure 17) that the final stress state near the projectile at different velocities at 4 μs is dominated by tensile stress. In the cratering stage, since Al_3_Ti has a yield strength of 1098 Mpa at a high strain rate while Ti has a yield strength of 800 Mpa, the Ti layer is extruded and fails under the combined action of the projectile and Al_3_Ti. Subsequently, the Al_3_Ti in front of the projectile is compressed and fragmented by the compressive stress wave and shock wave, and its performance is that the shear stress of the element is 0. After the Al_3_Ti element is deleted, different Ti layers are in direct contact, and the Ti layer is in direct contact with the Al layer. The Ti and Al layers produce compressive and shear plastic deformation under the action of the compressive stress wave load, which is consistent with the research of Cao [28], Wang [30], and Zelepugin [13]. At a position far from the projectile, the Ti, Al layers, and Al_3_Ti layer show a tensile stress state under the superposition of the reflected tensile wave and compressive wave. In addition, at this stage, it can be seen from Figure 8 that the delamination caused by the longitudinal crack propagation of the Ti-Al_3_Ti MIL composite mainly occurs on the back surface of the Ti-Al_3_Ti MIL target under the action of the tensile stress wave. In fact, this delamination damage is similar to the “spalling” feature of a large block material target under the action of a shock wave load [27]. This phenomenon can also be explained by the wave propagation theory: as is well known, the propagation velocity of the wave in the composite is much greater than the penetration velocity of the projectile. Therefore, when the stress wave reaches the back surface of the target, the penetration depth of the projectile is still very small. Due to different penetration velocities, the actual time for the stress wave to reach the free surface at the back of the target is 2–4 μs, which is obtained by subtracting the flight time of 0.9 μs when the projectile contacts the target from the time when the stress wave reaches the back free surface of the target, which is about 3–5 μs as is known from Figure 17. At this time, a tensile wave represented by a positive stress value and a compressive wave represented by a negative stress value can also be detected in each layer in the post-processor, indicating that the stress wave has reached the back of the target. In this short time, the compressive wave and the reflected tensile wave are superimposed, and the target shows a tensile stress state, as shown in Figure 17. Compared with the ductile Ti and Al, the brittle Al_3_Ti has lower tensile and shear strengths, so the reflected tensile wave causes the fragmentation of Al_3_Ti.

**Figure 18:**
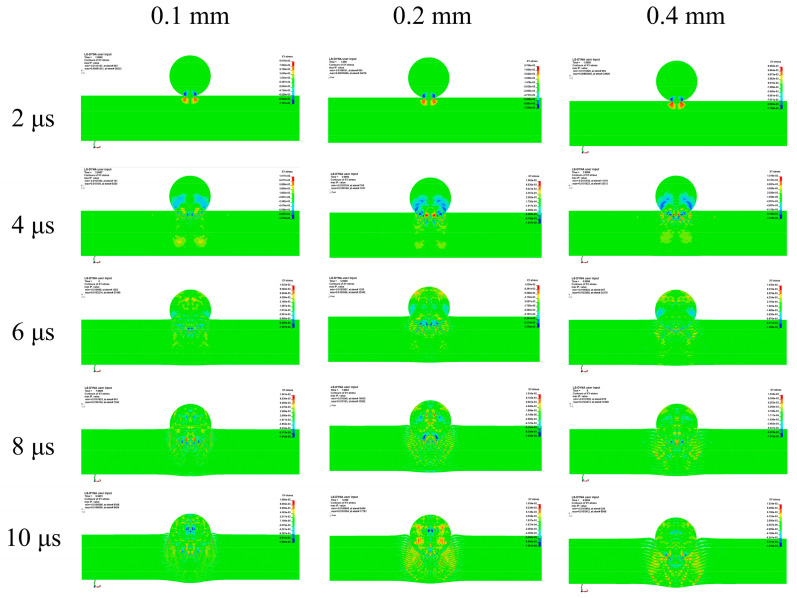
XY-plane stress distributions of a projectile with a penetration velocity of 600 m/s penetrating different configurations of Ti-Al_3_Ti targets at 2–10 μs.

To study the influence of the layer thickness on the stress wave characteristics of the target, we obtained the XY stress distributions of the Ti-Al_3_Ti laminated composites with different Ti layer thicknesses at 2–10 μs through the post-processing software, as shown in Figure 18. Among them, at 4–10 μs, with the increase in the Ti layer thickness, the stress distribution in the target changes from a stress concentration at 35° to a more circular diffuse distribution with a larger load action range. Each layer has more Al_3_Ti to absorb the impact load, and the maximum stress value at the front of the projectile at the same time gradually decreases. This is because the increase in the Ti layer thickness changes the reflection frequency of the stress wave, reduces the intensity and velocity of the superimposed stress wave, and changes the maximum stress value and distribution form.

In addition, in the stable penetration stage, the increase in the Ti layer thickness improves the flexural and shear strengths of the Ti layer, the transverse deformation changes from bending and stacking to bending and shearing as shown in Figure 11, and the transverse stress transfer is enhanced, thereby improving the anti-penetration performance of the target, as shown in Figure 18 (10 μs). In general, a thick matrix can better absorb and disperse the energy of the reflected wave, and an increase in the Ti layer thickness can improve the anti-penetration performance of the target by changing the stress distribution form and reducing the stress intensity, which is not achieved by a thin matrix.

### 3.5. Crack Propagation and Damage Evolution

**Figure 19:**
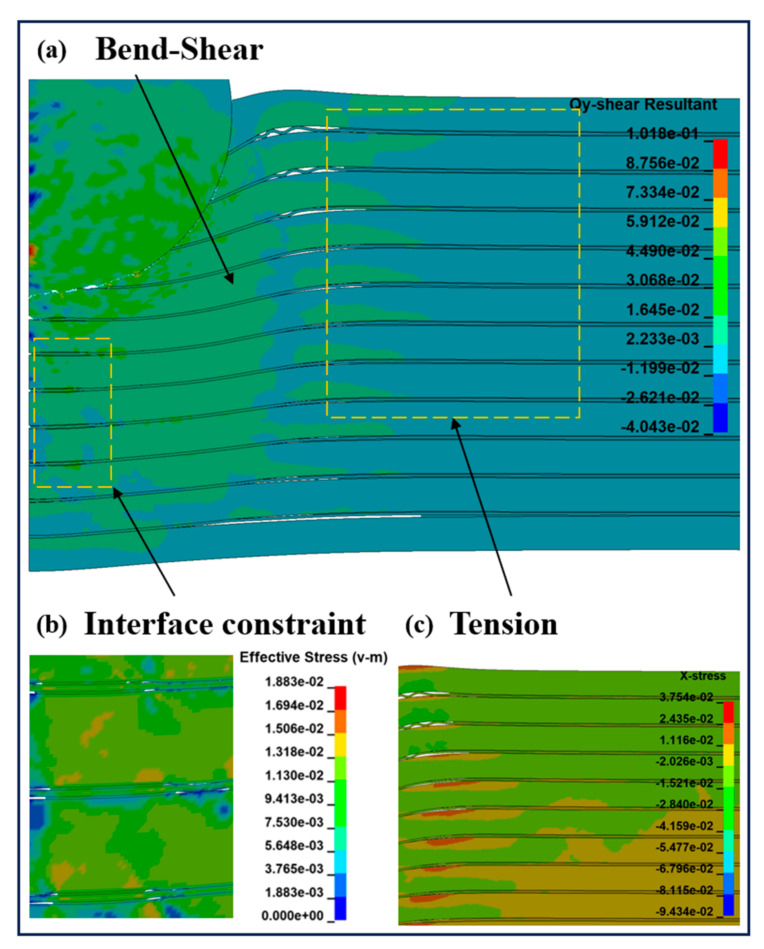
Schematic diagrams of the target under load during the penetration process: (**a**) bending-shearing; (**b**) interface constraint; (**c**) X-direction tension.

In numerical simulations, the finite element software characterizes the crack propagation in the target by making the failed elements invisible in the post-processing based on a reasonable constitutive model. Based on this principle, we studied the various damage mechanisms and crack propagations of the MIL composite formed under the combined action of tensile and compressive stress wave loads. It can be seen in Figure 19 that during the penetration process, the Ti-Al_3_Ti MIL composite target mainly hinders the penetration of the projectile in the following ways: the shear and flexural strengths of the ductile metal layer, the lateral tension after the stacking of the ductile layer, and the constraint on Al_3_Ti to increase the heterogeneous interface strength. Compared with traditional ductile and brittle metals, the hindering effect of this MIL composite on the projectile is complex and effective. The previous research results show that the contribution of this complex anti-penetration effect to the anti-penetration performance of the target is affected by the thickness of the Ti layer and exhibits different crack propagation modes. It should be noted that the 2D numerical model used in this paper can only provide a quantitative description of the crack extension of the target plate in this XZ-plane, which has some error in accuracy with the actual 3D crack extension.

**Figure 20:**
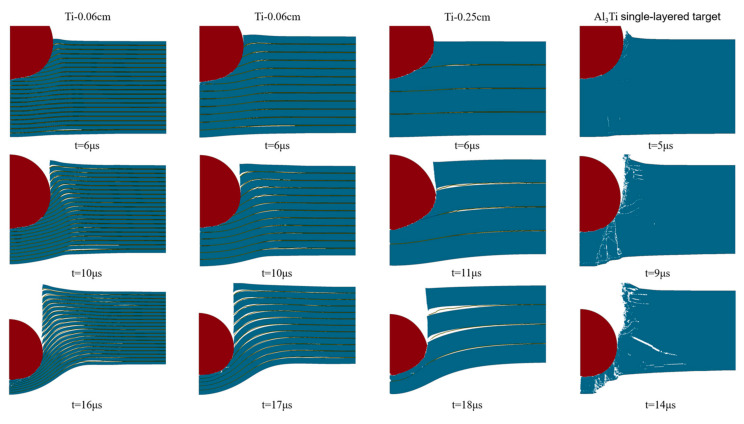
The deformation sequence of projectiles penetrating target plates with different configurations.

Figure 8 and Figure 20 show the damage evaluations of different configurations of MIL composite targets penetrated by tungsten alloy projectiles with an impact velocity of 1000 m/s. Obviously, the crack initiation, propagation, morphology, distribution, and delamination (spalling) can be analyzed according to these simulated images collected at different times. In Figure 20, we can see that when the projectile penetrates the MIL composite target, the projectile is blunted and damaged, and with the increase in the Ti layer thickness, the damage and blunting phenomena of the projectile are aggravated, and the velocity of the projectile is significantly reduced. This indicates that the thick Ti matrix significantly increases the penetration resistance of the projectile, especially in the initial and middle stages of penetration (approximately before 11 μs).

**Figure 21:**
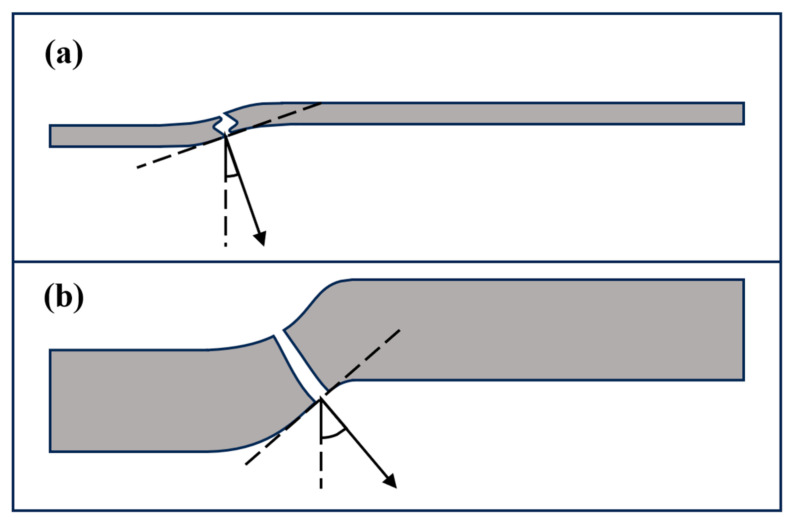
Schematic diagrams of the stress propagation directions of Ti layers with different thicknesses. (**a**) Thin matrix, (**b**) Thick matrix.

From the performance of the target, compared with the single-layer Al_3_Ti target, the crack propagation modes of the MIL composite target during the penetration process are failure along the thickness direction and transverse delamination, respectively. Additionally, with the increase in the Ti layer, the transverse delamination is significantly enlarged, and the longitudinal crack deflection to the transverse direction is accelerated. This phenomenon is caused by the following reasons: 1. The thickening of the Ti layer causes the transverse stress propagation in the layer to be farther and the heterogeneous structure failure to be more severe. 2. The tension and shear generated by the warping of the Ti layer aggravate the interlayer crack propagation of the target. 3. The Ti layer changes the stress transfer direction through different bending angles, as shown in Figure 21. In general, the difference in crack propagation modes has a very important influence on the anti-penetration performance of the target, especially in the initial stage of penetration. The increase in the thickness of the Ti layer can make more heterogeneous structures in the target hinder the penetration of the projectile by changing the crack propagation direction.

### 3.6. Energy Behavior of Ti-Al_3_Ti Target with Different Ti Thicknesses

**Figure 22:**
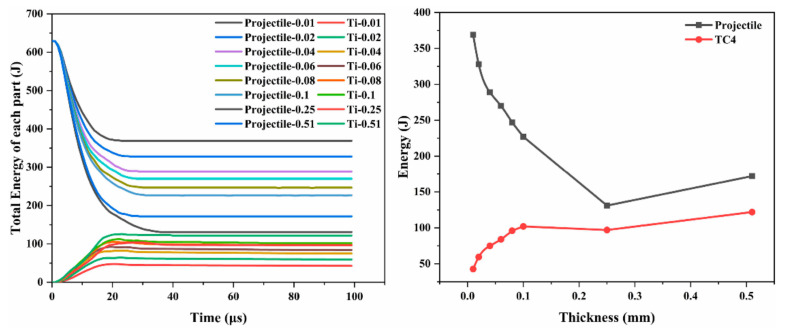
(**left**) Penetration energy–time history plot and (**right**) energy–thickness relations of the projectile and Ti layer in the Ti-Al_3_Ti target at a velocity of 1000 m/s.

The results in Figure 22 indicate that compared to the Ti-Al_3_Ti target with a lower thickness of Ti, the Ti-Al_3_Ti target with a large thickness of Ti showed a significant increase in the energy loss rate of the projectile and the total energy absorption rate of Ti during penetration and that it reached the maximum value at a Ti thickness of 2.5 mm. In addition, this part of the energy–thickness curve’s slope gradually decreases, and the relation is non-linear between the energy and the thickness. This relation is due to the excellent plasticity and strength of Ti, which intensify the blunting and damage of the projectile simultaneously, thus increasing the contact area and friction between the projectile and the target. Meanwhile, in these Ti-Al_3_Ti targets with different Ti thicknesses, the total energy of Ti gradually increases before 19 μs, fluctuates between ~19 and 29 μs, and then gradually decreases. After penetrating the Ti-Al_3_Ti target, the energy is gradually released. However, for the Ti-Al_3_Ti targets with higher thicknesses of Ti, the maximum absorbable energy of the target is significantly increased. The reasons for the energy changes are as follows: as the penetration process progresses, due to the continuous friction between the target and the projectile, influenced by dynamic and static friction, the energy of the Ti layer continues to change between ~19 and 29 μs. At the same time, the release of energy is caused by the loss of HDI strengthening and strain hardening after the failure of Al_3_Ti. Both of these states result in the total energy of Ti being in a dynamic fluctuating state. Additionally, due to the extensive failure of Al_3_Ti and the plastic deformation of Ti, the internal energy of Ti gradually decreases. The difference in the peak total energy of Ti is positively correlated with the content but shows a nonlinear relationship.

## 4. Conclusions

In this work, a impact experiment and numerical simulations were used to study the effects of the Ti thickness on the penetration resistance behavior and performance of Ti-Al_3_Ti targets. The projectile residual velocity, penetration process, crack propagation, and energy variation of the Ti-Al_3_Ti targets with thicknesses of 0.1 mm, 0.2 mm, 0.4 mm, 0.6 mm, 0.8 mm, 1 mm, 2.5 mm, and 5.1 mm at a velocity of 1000 m/s were analyzed emphatically. The following key conclusions can be drawn.

The simulation results of the projectile residual velocity are consistent with the experimental results. The penetration velocity range of the projectile in the numerical simulation is 400–1000 m/s. There is a nonlinear relationship between the residual velocity of the projectile and the thickness of the Ti layer. The simulation shows that, compared with the 0.1 mm Ti layer, the 2.5 mm Ti layer reduces the residual velocity of the projectile by 100% (600 m/s), 72% (800 m/s) and 38.5% (1000 m/s), and the mass of the target plate only increases by 0.1 g. The optimal thickness of the Ti layer in the Ti-Al_3_Ti MIL composite is 2.5 mm, and the residual velocity of the projectile first increases and then decreases with the increase in the thickness of the Ti layer.

For the Ti-Al_3_Ti MIL composite target plates with different Ti layer thicknesses, the Ti layer with a thickness of millimeters can more effectively improve the anti-penetration performance of the target in terms of load transfer and deformation mode compared with the traditional micron-level thickness. When the total thickness difference of the Ti-Al_3_Ti MIL composite target plate is 0.1 mm, for the 0.8 mm and 2.5 mm Ti layers, the stresses of Al_3_Ti at the same position increase by 15% and 24%, respectively, and the energy of the projectile decreases by 33% and 65%, indicating that as the Ti layer thickens, the impact load absorbed by Al_3_Ti is enhanced. The target with a millimeter-level Ti layer improves the anti-penetration performance of the target by hindering the early failure of the local heterogeneous structure, enhancing the interlayer coordination ability of the target in the early and middle stages of penetration, increasing the content of the material involved in hindering the projectile, and increasing the contact time between the target and the projectile.

Furthermore, during the propagation of the stress wave, the Ti with a millimeter-level thickness can improve the anti-penetration performance of the target material on the basis of the anti-penetration behavior of the Ti-Al_3_Ti MIL composite target with a micron-level Ti thickness by changing the form of the stress distribution. The increase in the thickness of the Ti layer changes the crack propagation direction of the Ti-Al_3_Ti MIL target. Based on the crack propagation angle when the thickness of the Ti layer is 0.4 mm, the crack propagation angles when the thickness of the Ti layer is 0.6 mm and 2.5 mm increase by 4° and 14°, respectively. This results in the penetration of the projectile being hindered by more heterogeneous structures. The Ti target material with a millimeter-level thickness causes more significant damage to the projectile in the cratering stage and stable penetration stages of penetration, indicating that the thick Ti layer absorbs a certain amount of energy through plastic deformation, maximizing the effective area of the heterogeneous deformation-induced (HDI) strengthening.

In general, the anti-penetration performance of the Ti-Al_3_Ti MIL composite target with a millimeter-level Ti layer thickness has been improved in many aspects. However, since the model is a 2D model, it can only accurately describe the crack propagation trend of the Ti-Al_3_Ti MIL composite target plate in the cut plane. There are certain limitations in the results compared with the 3D numerical simulation with a large number of meshes. In future work, it is necessary to optimize the 3D simulation through numerical methods to obtain more accurate results. In this paper, the vertical penetration of the projectile into the target plate is studied. We will study numerical simulations of variable impact trajectories and simultaneous multiple impacts in future work to make up for the shortcomings of the current research. In addition, by reducing the size limitations of the raw materials, the technical cost of mass-producing high-performance impact-resistant materials can also be reduced.

## Figures and Tables

**Table 1:** Chemical composition of Ti-6Al-4V foil and Al foil [6,7,8].

Materials	Composition (wt.%)
TI foil	Ti: balance, Al: 5.50–6.80, V: 3.50–4.50, Fe ≤ 0.30, O ≤ 0.20, C ≤ 0.10, N ≤ 0.05, H ≤ 0.01
Al foil	Al: 99.60, Fe: 0.35, Si: 0.25, Cu: 0.05, Zn: 0.05, V: 0.05, Mg: 0.03, Ti: 0.35

**Table 2:** Specific size parameters of the target model.

Thickness and Number of Al Layers (mm × *n*)	Thickness and Number of Al_3_Ti Layers (mm × *n*)	Thickness and Number of Ti Layers (mm × *n*)	The Element Number of the Target
0.06 × 51	0.02 × 102	0.1 × 52	219800

**Table 3:** Parameters of the J-C constitutive models for tungsten, Ti, and Al [6,7,8].

	ρ (g.cm^−3^)	E (GPa)	ν	A (GPa)	B (GPa)	c	m	*n*	T_Melt_ (K)
Al	2.70	135.00	0.33	989.00	70.00	0.00	1.00	0.37	300.00
Ti	4.428	113.00	0.342	1098.00	1092.00	0.014	1.10	0.93	1878.00
Wu	17.30	310.00	0.30	1506.00	177.00	0.016	1.10	0.12	1752.00

T_Melt_ is the melting point temperature of the material.

**Table 4:** Parameters of the JH-2 constitutive model for Al_3_Ti [6,7,8].

ρ (g.cm^−3^)	E (GPa)	v	A	B	C	M	*n*	T (GPa)
3.35	216.00	0.17	0.85	0.31	0.013	0.21	0.29	0.20
$P_{HEL}$ (GPa)	$D_{1}$	$D_{2}$	$K_{1}$(GPa)	$K_{2}$(GPa)	$K_{3}$(GPa)			
1.842	0.02	1.85	2.01	2.60	0.00			

**Table 5:** Comparison of experimental and simulation results. (“PP” indicates that the projectile did not penetrate the target).

Item	Initial Velocity (m/s)	Experimental Results (m/s)	Simulate Results (m/s)
1	364	PP	PP
2	388	PP	PP
3	485	PP	PP
4	533	162	157

**Table 6:** The results of simulations (“CP” indicates that the projectile completely penetrated the target, and “PP” is the converse).

Item		1	2	3	4	5	6	7	8	9
Ti Thickness (mm)		0.1	0.2	0.4	0.6	0.8	1	2	2.5	5.1
Initial velocity for 400 m/s	Residual velocity (m/s)	−4.65	−6.64	−9.21	−11.3	−14.4	−15.3	−16.3	−17.2	−17.1
Results		PP	PP	PP	PP	PP	PP	PP	PP	PP
Initial velocity for 600 m/s	Residual velocity (m/s)	300	150	4	0	−3.63	−2.54	−1.43	−5.21	0
Results		CP	CP	CP	PP	PP	PP	PP	PP	PP
Initial velocity for 800 m/s	Residual velocity (m/s)	550	503	445	410	358	319	253	150	160
Results		CP	CP	CP	CP	CP	CP	CP	CP	CP
Initial velocity for 1000 m/s	Residual velocity (m/s)	758	717	669	645	616	580	545	466	514
Results		CP	CP	CP	CP	CP	CP	CP	CP	CP

**Table 7:** Impedance and density of the three metals.

Material	Ti	Al_3_Ti	Al
Density [g·cm^−3^] $(g\cdot cm^{-3}$)	4.51	3.37	2.7
Wave velocity ($Km\cdot s^{-1})$	4.77	8.09	5.09
Wave impedance $\left(MPa\cdot m^{-1}\cdot s^{-1}\right)$	21.51	27.26	13.74

## Data Availability

The original contributions presented in the study are included in the article, further inquiries can be directed to the corresponding author.
